# Dr. Fly’s Letter, and What Has Come of It,—So Far

**Published:** 1909-05

**Authors:** 


### DR. FLY'S LETTER, AND WHAT HAS COME OF IT,—SO FAR.

In the April number of *The Texas State Journal of Medicine*, page 309, appeared an editorial saying in effect that that journal would always publish communications from reputable physicians, but not from men who are not in good standing in their county society and who do not subscribe to the code of ethics. This was a slur intended for Dr. A. W. Fly, a member of the Board of Regents of the University of Texas, who was appointed at the especial request of the Galveston County Medical Society, embracing the entire faculty of the State Medical College and indeed every reputable physician in the city and county.

It will be remembered that Dr. Fly had sent a letter to the *State Journal of Medicine,* respectfully requesting that it be published for the information of the profession, asking a number of pertinent questions of great interest to every physician. The editor made excuses for not publishing it in March, saying it was too late. It did not appear in April, and the slanderous editorial quoted above was intended to forestall its publication elsewhere. It appeared in full in the "Red Back" and the *Courier-Record of Medicine* for April, together with the correspondence between Dr. Fly and the editor. The editor evaded a reply to the inquiry whether the letter would appear in the April number by saying that the Trustees had not yet passed upon it [March 26]. Dr. Fly had information that the Trustees and the editor had rejected the letter *as early as March 3,* thus nailing an untruth. Dr. Fly demanded the return of his manuscript, which was refused. He then wrote Dr. Chase to send it instanter, or he would "come after it." The editor then requested Galveston County Medical Society, for his safety, to have Dr. Fly put under peace bonds—else, he thought, it would not be safe to attend the Galveston meeting this month.

It seems that then Dr. J. S. Lankford, one of the Trustees of the State Association Journal of Medicine, had the editor to return the paper, and Dr. Fly acknowledged it in the following letter:

GALVESTON, TEXAS, April 26, 1909.

*Dr. J. S. Lankford, President Board of Directors [Trustees], Texas State Journal of Medicine, San Antonio, Texas.*

DEAR DOCTOR: I am in receipt of the manuscript that has been the cause of an unpleasant controversy.

I care absolutely nothing for the opinion of Dr. Chase. Had he treated me fairly in the outset and told me the truth, instead of evading me as he did, and retaining my manuscript, there would have been nothing to regret on either side.

I realize that Drs. Thompson, Redd and yourself are gentlemen of the old school. I do not know Dr. Sturgis, therefore am not in a position to give expert opinion on that subject.

How any gentleman can for a moment consider my letter to the editor of the Texas Journal as "anonymous," is something beyond my conception. Any insinuation to the effect that I am acting in the interest of the proprietary people, either directly or indirectly, is as false as it is unjust.

The mere fact that you gentlemen concur in the opinion of Dr. Chase shows that my designation, "autocratic," was not out of place. Very respectfully,

A. W. FLY.

How puerile and impotently spiteful was the editorial intimating that Dr. Fly was not in good standing is demonstrated in the following,—not that any defense or showing of the kind is necessary, but to show how our editor will resort to such contemptible methods to wound those who do not bow down and worship, as he does, the detested Simmons.

David H. Lawrence, President. James J. Terrill, Secretary.

GALVESTON COUNTY MEDICAL SOCIETY
Galveston, Texas

*Dr. A. W. Fly, Galveston, Texas.*

DEAR DR. FLY: At the request of Dr. Lawrence, I am sending you a copy of the proceedings of the Galveston County Medical Society, with reference to your appointment as local regent. If there is any other thing I can do in this regard, please command me. Very sincerely yours,

JAMES J. TERRILL,

April 20th, 1909. Secretary.

At a meeting of the Galveston County Medical Society held January 20, 1909, a motion was carried that the Galveston County Medical Society unqualifiedly indorse Dr. A. W. Fly for appointment by the Governor as Local Regent for the University of Texas. This motion was amended so that the Secretary and Dr. H. P. Cooke be instructed to draw up this motion in due form for transmission to the Governor. "After many enthusiastic speeches paying glowing tribute to Dr. Fly's ability, his personality and his fitness for the appointment, the motion was unanimously carried." (Extract from the minutes of this meeting.)

In accordance with the above instructions, the following letter was sent to Governor Campbell:

*"To His Excellency, Governor Thomas M. Campbell, Austin, Texas.*

"DEAR SIR: At a meeting of the Galveston County Medical Society, held January 20th, 1909, the following motion was unanimously adopted:

" 'It is hereby moved that the Galveston County Medical Society do unqualifiedly indorse Dr. A. W. Fly for appointment by the Governor as Local Regent for the University of Texas.'

"Your own acquaintance with Dr. Fly, the man, the citizen, and the doctor, renders it unnecessary for us to enter into a lengthy discussion of his qualifications or of his fitness for this appointment. Of this you are already aware.

Very sincerely yours,

(Signed) "D. H. LAWRENCE,
"President Galveston County Medical Society.

(Signed) "JAMES J. TERRILL,
"Secretary Galveston County Medical Society.

"January 22nd, 1909."

The above is a true and correct copy of the proceedings of the Galveston County Medical Society in the matter of indorsing Dr. A. W. Fly for appointment as Local Regent of the University of Texas.

JAMES J. TERRILL,
Secretary Galveston County Medical Society.

April 20th, 1909.

I attach herewith a copy of the petition to the Governor to appoint Dr. Fly a Regent, which was done, and all Texas is honored by the appointment.

"GALVESTON, TEXAS, January 16, 1909.

*"To His Excellency, Governor T. M. Campbell, Austin, Texas.*

"SIR: We, the undersigned physicians of Galveston, recognizing the great administrative and executive ability and high professional attainments of our fellow practitioner, Dr. A. W. Fly, do most earnestly indorse him as highly qualified to fill the position of Regent of the University of Texas. It is very evident that it would be of great advantage to have a strong local medical representative on the Board, to assist in the administration of the Medical Department of the University.

"Most respectfully yours,"

[Signed by the President, the Dean and all the Medical Faculty of the Medical Department of the University, all the health officials, and the leading practitioners of the city and county.]

Comment is unnecessary. Will the State Association—will the Trustees countenance such mud slinging?

---

ANNUAL TRANSACTIONS VS. A JOURNAL.—Now and then we hear of some who would rather have an annual volume of transactions than the present monthly *State Journal.* These persons we are prepared to accommodate. Each member now pays $2.00 through his county secretary, which entitles him to membership in the State Association and to a copy of its publication. Any member who wishes may obtain an annual bound volume of the year's *Journal* by sending his order and an extra $1.50 at the beginning of the year. Any one at the end of the year who wishes his volume bound may send $1.50 with the unbound copies and receive a volume bound in half morocco by return mail. The old volume of Transactions cost $5.00, had half the reading matter, contained no current medical news, and the scientific papers—well, if you want to realize the advance in the scientific work of the Association since its re-organization, compare the average paper in the old volume of Transactions with the average original paper now published in this *Journal.—Texas State Journal of Medicine.*

The statement that the Transactions in book form cost $5.00 per copy is the wildest and most reckless guess the editor of the *State Journal of Medicine* ever made, and he seems to be given to guessing. The books of Von Boeckmann-Jones Company, the printers, were accessible to him, and he might have obtained the exact figures, as I have done, and saved himself from the suspicion of purposely misstating the cost.

I take the last two volumes issued, 1903 and 1904. They are the largest and handsomest volumes ever issued for the Association. They are printed with new Ronaldson type, on heavy calendered paper, deckel edge, and handsomely bound in cloth and gold lettered, and contain, each volume, over 600 pages.

Of 1903, 650 copies were issued. Total cost, delivered, including wrapping, postage, expressage prepaid and ten presentation copies de luxe for the officers, was $972.97, exactly $1.49 per volume.

Of 1904, there were issued 2550 copies, 645 pages, uniform with preceding volumes. They cost, delivered, postage, expressage, extra de luxe copies, wrapping,—every item of expense, $1968.67—exactly 77 cents per volume. Comment is unnecessary. A copy of each of the above will be on exhibition at the Galveston meeting.

Why not resume publication of an annual volume of Transactions, in addition to the monthly journal? The majority desire it and should be heard on a proposition of this kind. In an annual volume, strongly bound, there should be preserved the original papers read at each meeting, the discussions, etc., in permanent form. Only in this way can they be preserved. The cost, as has just been shown, is inconsiderable—77 cents per volume. The twelve copies of the *Journal* are preserved by few, and bound by almost none, and if bound there is so much extraneous matter, worthless for preservation, that it is not worth while to go to the expense of binding. In after years there will be found to be a hiatus or gap in the record, just as there is now between 1877 and 1885—journalism was tried about eight years, and no copy of the journal for those years has been preserved. The monthly issue of the *Journal* could be continued, as a bulletin, or newspaper of the Association, without diminishing its revenue in the least. Under the present system the *Journal* has been conducted at a great profit, and there are piled up in the bank several thousand dollars lying idle or out at interest. At Corpus Christi the Trustees reported a surplus on hand, all expenses paid, of $8720.45.* This at the end of four years. At the Galveston meeting this sum will be shown to have grown approximately to $10,000. In other words an excess of $2000 a year for five years will have been taken unnecessarily from the members and should go back to them in some shape. That would give each member, if paid in cash, about $3. What better use can be made of it than putting it in a yearly volume for permanent record in the archives? What is going to be done with this surplus?

*See *Texas State Journal of Medicine*, June, 1908, p. 39.

